# Diagnostic performance of metagenomic next-generation sequencing for the detection of pathogens in cerebrospinal fluid in pediatric patients with central nervous system infection: a systematic review and meta-analysis

**DOI:** 10.1186/s12879-024-09010-y

**Published:** 2024-01-18

**Authors:** Sike He, Ying Xiong, Teng Tu, Jiaming Feng, Yu Fu, Xu Hu, Neng Wang, Dapeng Li

**Affiliations:** 1 West China School of Medicine, Sichuan University, Chengdu, China; 2 Center of Infectious Diseases, West China Hospital, Sichuan University, Chengdu, China; 3 Department of Periodical Press/Chinese Evidence-Based Medicine Center, West China Hospital, Sichuan University, Chengdu, China; 4 Department of Urology, West China Hospital, Sichuan University, Chengdu, China; 5 Key Laboratory of Drug-Targeting and Drug Delivery System of the Education Ministry and Sichuan Province, Sichuan Engineering Laboratory for Plant-Sourced Drug, Sichuan Research Center for Drug Precision Industrial Technology, West China School of Pharmacy, Sichuan University, Chengdu, China

**Keywords:** Pediatric infection, Central nervous system infection, Metagenomic next-generation sequencing, Diagnostic performance, Meta-analysis

## Abstract

**Background:**

Detecting pathogens in pediatric central nervous system infection (CNSI) is still a major challenge in medicine. In addition to conventional diagnostic patterns, metagenomic next-generation sequencing (mNGS) shows great potential in pathogen detection. Therefore, we systematically evaluated the diagnostic performance of mNGS in cerebrospinal fluid (CSF) in pediatric patients with CNSI.

**Methods:**

Related literature was searched in the Web of Science, PubMed, Embase, and Cochrane Library. We screened the literature and extracted the data according to the selection criteria. The quality of included studies was assessed by the Quality Assessment of Diagnostic Accuracy Studies-2 (QUADAS-2) tool and the certainty of the evidence was measured by the Grading of Recommendations, Assessment, Development, and Evaluations (GRADE) score system. Then, the pooled sensitivity, specificity, positive likelihood ratio (PLR), negative likelihood ratio (NLR), diagnostic odd’s ratio (DOR), and area under the curve (AUC) of the summary receiver operating characteristic curve (sROC) were estimated in Stata Software and MetaDisc. Subgroup analyses were performed to investigate the potential factors that influence the diagnostic performance.

**Results:**

A total of 10 studies were included in the meta-analysis. The combined sensitivity was 0.68 (95% confidence interval [CI]: 0.59 to 0.76, *I*^2^ = 66.77%, *p* < 0.001), and the combined specificity was 0.89 (95% CI: 0.80 to 0.95, *I*^2^ = 83.37%, *p* < 0.001). The AUC of sROC was 0.85 (95% CI, 0.81 to 0.87). The quality level of evidence elevated by the GRADE score system was low.

**Conclusions:**

Current evidence shows that mNGS presents a good diagnostic performance in pediatric CNSI. Due to the limited quality and quantity of the included studies, more high-quality studies are needed to verify the above conclusion.

**Supplementary Information:**

The online version contains supplementary material available at 10.1186/s12879-024-09010-y.

## Introduction

Pediatric central nervous system infection (CNSI) is the main cause of death in children, with high mortality and morbidity and poor prognosis [1, 2]. Because of the various pathogens, occult onset, atypical clinical symptoms, and rapid progression, pediatric infectious diseases are difficult to diagnose, which may lead to high mortality [2, 3]. Identifying pathogens is vital for both therapy and prognosis [4, 5]. Conventional etiology detection (such as culture and smears) is the gold standard but has a low positive rate, and it may take a long time [4, 6]. Previous studies indicated that the pathogens were not detectable in approximately 60% of cases after using comprehensive testing methods [7]. Metagenomic next-generation sequencing (mNGS), also called shotgun sequencing, based on high-throughput sequencing technology, is a burgeoning unbiased pathogen detection method [8, 9]. Its advantages include high throughput, wide coverage, high accuracy, and efficiency. Hence, it has been successfully applied in the diagnosis and treatment of difficult and critical infectious diseases, identification of unknown pathogens, drug resistance gene monitoring, epidemiological tracking investigation, etc. mNGS can significantly increase the positive rate of detection, especially in *Listeria monocytogenes*, *Mycobacterium tuberculosis*, nontuberculous mycobacteria, *Nocardia* spp., and other viruses and fungi with a low positive rate of culture [10–12]. In 2014, it was first applied to detect *Leptospira Santarosai* after a period of 4 months of no special diagnosis [13]. Currently, mNGS is widely used for the diagnosis of pediatric infection. However, the studies associated with the clinical application of mNGS are mainly case reports and some small-scale cohort studies [14–16]. The clinical diagnostic performance of mNGS in pediatric CNSI remains to be evaluated. Therefore, we performed this systematic review and meta-analysis of mNGS for diagnosing CNSI, including a comprehensive and systematic analysis of its diagnostic performance. We aim to provide reliable evidence for the application of mNGS in diagnosing pediatric CNSI.

## Methods

This is a systematic review and meta-analysis for diagnostic test accuracy. We followed the Preferred Reporting Items for Systematic Reviews and Meta-Analyses (PRISMA) reporting guidelines [17]. The study protocol has been registered in the International Prospective Register of Systematic Reviews (PROSPERO) (Registration number: CRD42023393769).

### Selection criteria and search strategy

We included original studies such as case–control, retrospective cohort, and prospective cohort studies that assessed the efficacy of mNGS in pediatric CNSI. The enrolled patients must have a clear definition of CNSI. The diagnostic criteria of CNSI mainly consist of clinical symptoms, imaging evidence, and laboratory tests, and all the final diagnoses were confirmed by professional clinicians.

According to the definition of the Centers for Disease Control, the diagnostic criteria for CNSI mainly include a) one of the following clinical signs occurring without a clear cause: fever (> 38 °C), stiff neck, headache, meningeal signs, cranial nerve signs, confusion or changing of consciousness; b) elevated white cells, protein and/or decreased glucose in CSF; and c) identification of pathogens in CSF [18].

The inclusion criteria were as follows: a) case–control or cohort studies that reported the diagnostic measurements (true positive [TP], false positive [FP], true negative [TN], and false negative [FN]); b) the pathogen detection methods included mNGS; c) the participants consisted of at least 10 pediatric samples; d) the gold standard is the combination of clinical diagnosis and traditional pathogen detection methods (culture, smear. etc.). The exclusion criteria were as follows: a) duplicated literature, case reports, comments, editorials, meeting abstracts, and reviews; b) the detailed methods of mNGS were not clearly described; c) the gold standard was unclear; d) the TP, FP, TN, and FN data could not be obtained; and e) the sample size was less than 10 participants.

A literature search was performed using Web of Science, Embase, Medline (via PubMed), and Cochrane Library. There were no limitations set on the date (from inception to January 29, 2023) or language to ensure that more studies were included. After removing duplicated literature by using EndNote, two reviewers (HS, FJ) independently screened the literature through a title/abstract screening and then a subsequent full-text screening, and disputes were resolved through negotiation with the third reviewer (LD). The complete search strategy was as follows: (mNGS OR metagenomic next-generation sequencing OR metagenomic next generation sequencing OR metagenomic sequencing OR shotgun metagenomic) AND (central nervous system infection OR CNS infection OR encephalitis OR meningitis) AND (children OR pediatric OR paediatric OR neonatal OR infant).

### Data extraction

The following data from the individual studies were extracted: (a) basic characteristics, including first author, publication year, and area; (b) the type of study and sample size; (c) the methodological quality, the gold standard for diagnosis of CNSI, and criteria for a positive mNGS result; (d) mNGS sequencing technology, mNGS sequencing method, and mNGS sequencing conditions (sequencing platform, DNA/RNA extraction, and bioinformatics analysis); and (e) the diagnostic accuracy measurements. For the extraction of sensitivity, the research subject was a patient diagnosed with CNSI by clinicians. Patients with CNSI have corresponding clinical manifestations, laboratory indicators, and imaging manifestations. Notably, when extracting the original specificity value, the research subject must be a healthy individual or a patient with a precise diagnosis of non-CNSI. Two researchers (HS and FJ) independently extracted these data, and disagreements between the two reviewers during the data extraction process were resolved by consensus with the third author (HX).

### Quality assessment and appraisal of certainty of evidence

Three authors (HS, FJ, and TT) independently performed a quality assessment of all included studies, and disagreements were resolved by discussion. The quality of the included studies was assessed using the Quality Assessment of Diagnostic Accuracy Studies (QUADAS)-2 tool [19] (Review Manager, version 5.3, The Nordic Cochrane Centre, The Cochrane Collaboration and Copenhagen, 2014). The Grading of Recommendations, Assessment, Development, and Evaluations (GRADE) system was applied to evaluate the quality of evidence (GRADEpro GDT software, via https://www.gradepro.org) [20, 21].

### Data analysis

Statistical analysis was performed using Stata (version 16.0) software. The diagnostic sensitivity, specificity, positive likelihood ratio (PLR), negative likelihood ratio (NLR), and diagnostic odds ratio (DOR) of mNGS were calculated by a random-effects model or a fixed-effects model. The heterogeneity among the studies was assessed using the chi-square test and the *I*^2^ statistic. We also plotted summary receiver operating characteristic (sROC) curves for studies reporting both sensitivity and specificity and calculated the results. To further analyse the potential factors that may affect heterogeneity, subgroup analyses, leave-one-out analysis, and meta-regressions were performed according to continent, research type, study direction, whether patients were immunocompromised or severely ill status, sample method, pathogen type, and sequencing platform. A two-tailed *p-*value of < 0.05 was considered statistically significant. *P* < 0.10 or *I*^*2*^ > 50% was considered statistically significant for heterogeneity. Significant heterogeneity was pooled using a random-effects model. Stata software (version 16.0) and MetaDisc (version 1.4) were used for statistical analyses.

## Results

### Literature search results

**Fig. 1:**
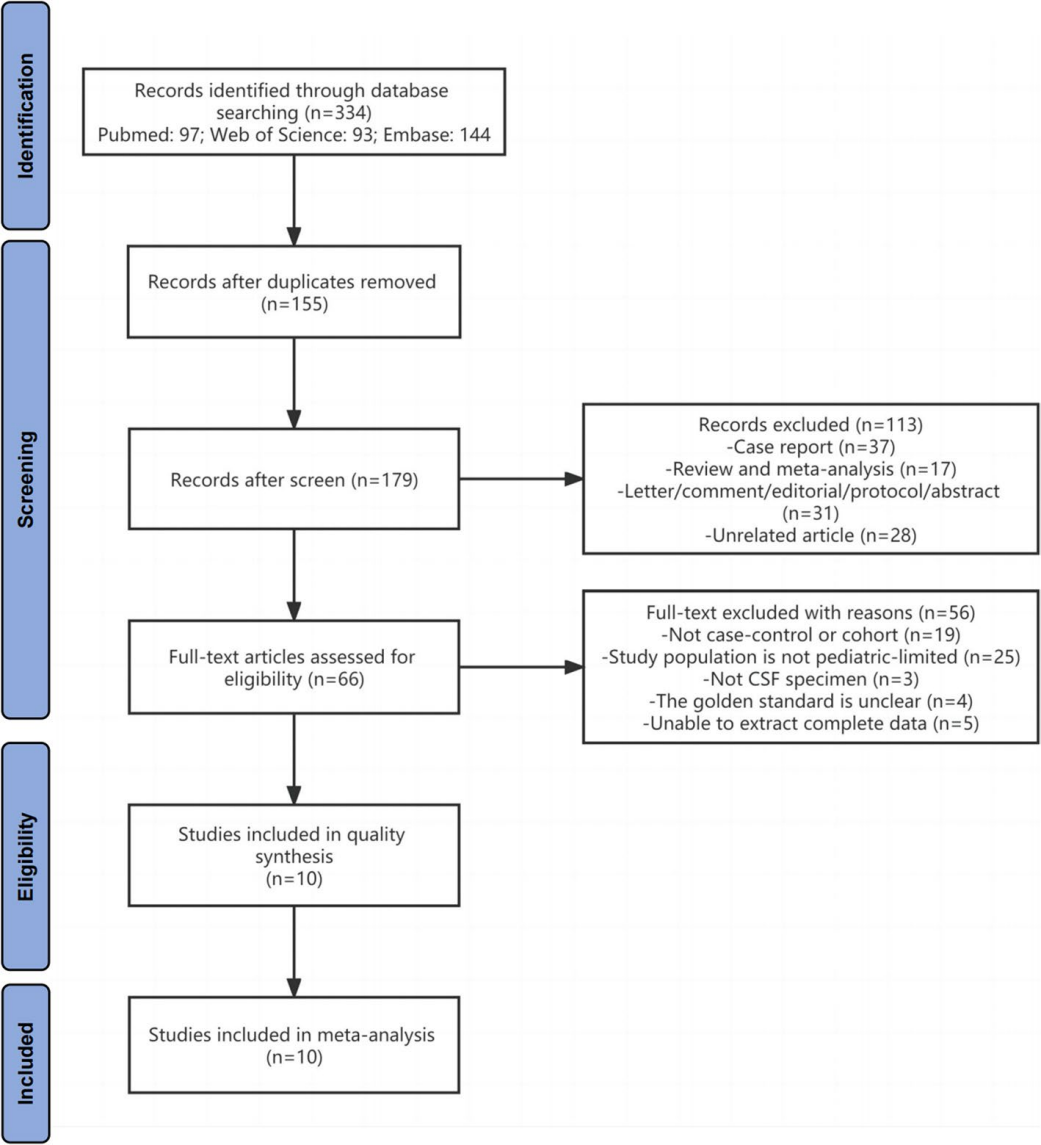
Flow chart of study retrieval

This search strategy identified 334 studies, and 10 fulfilled all selection criteria to be included in the meta-analysis [4, 22–30]. The details of the literature search and screening process are shown in Fig. 1. The basic characteristics of the 10 included studies are listed in Table 1.

**Table 1:** Characteristics of the included studies

Study name (author, year)	Research Type	Country/Continent	Samples	Study design	Multi/single-center	Sequencing platform	Patients were severely ill or immunocompromised	Sample pattern	Pathogen type	TP	FP	FN	TN
(Leon, 2018) [24]	Cohort	Spain/Europe	20	Retrospective	Single	Illumina	No	Frozen	isolated	10	0	10	0
(Saha, 2019) [28]	Case–control	Bangladesh/Asia	91	Retrospective	Single	Illumina	No	Fresh	mixed	35	0	11	45
(Zhang, 2019) [4]	Case–control	China/Asia	135	Retrospective	Single	BGISEQ	No	Fresh	isolated	32	6	11	86
(Haston, 2020) [23]	Cohort	USA/America	20	Prospective	Single	Illumina	No	Fresh	mixed	3	3	0	14
(Leon, 2020) [25]	Cohort	Spain/Europe	20	Retrospective	Single	Illumina	No	Fresh	isolated	8	0	12	0
(Ge, 2021) [22]	Cohort	China/Asia	101	Prospective	Single	Illumina	No	Fresh	mixed	5	15	0	81
(Guo, 2021) [29]	Case–control	China/Asia	17	Retrospective	Single	BGISEQ	No	Fresh	isolated	11	0	4	2
(Ramchandar, 2021) [21]	Cohort	USA/America	70	Prospective	Multi	Illumina	No	Frozen	mixed	20	4	5	41
(Chen, 2022) [30]	Cohort	China/Asia	127	Prospective	Single	Illumina	No	Frozen	isolated	32	23	27	45
(Qu, 2022) [26]	Cohort	China/Asia	15	Retrospective	Single	BGISEQ	Yes	Frozen	mixed	10	1	1	3

### Quality assessment and certainty of evidence

**Fig. 2:**
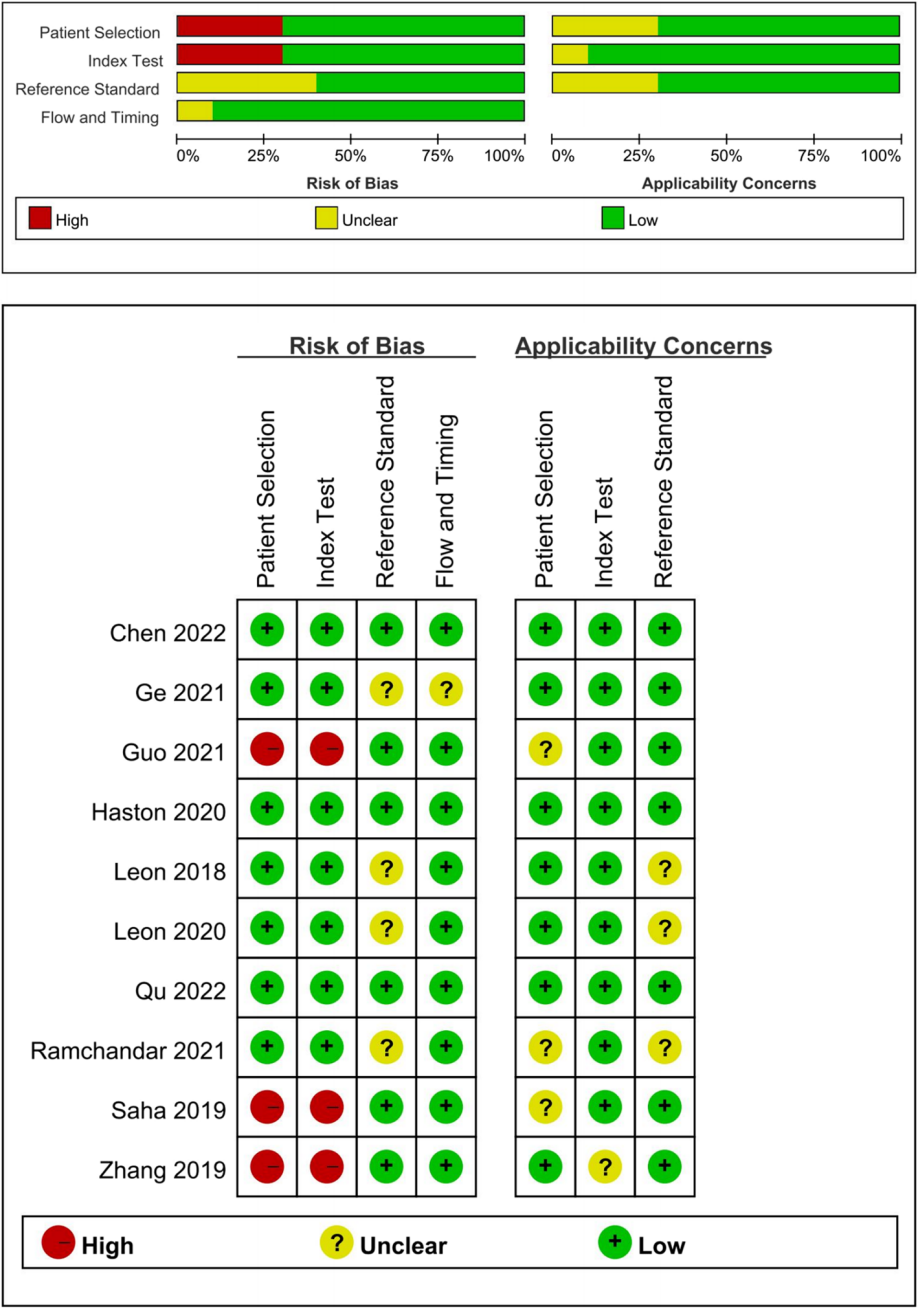
Quality assessment of the included studies by the QUADAS-2 tool

The quality of studies was assessed by the QUADAS-2 tool. Most of the studies were high quality with a low risk of bias and low applicability concerns, and the details of each study are shown (Fig. 2). The main source of risk of bias was patient selection, index test [4, 28, 29], and the description of reference test also contributed to the risk [22, 24, 25, 27]. Furthermore, patient selection and reference standards also primarily impact the applicability concerns [24, 25, 27–29]. The certainty of the evidence was evaluated to be low by the GRADE score system (Supplementary Fig. 1).

### Diagnostic performance of mNGS

**Fig. 3:**
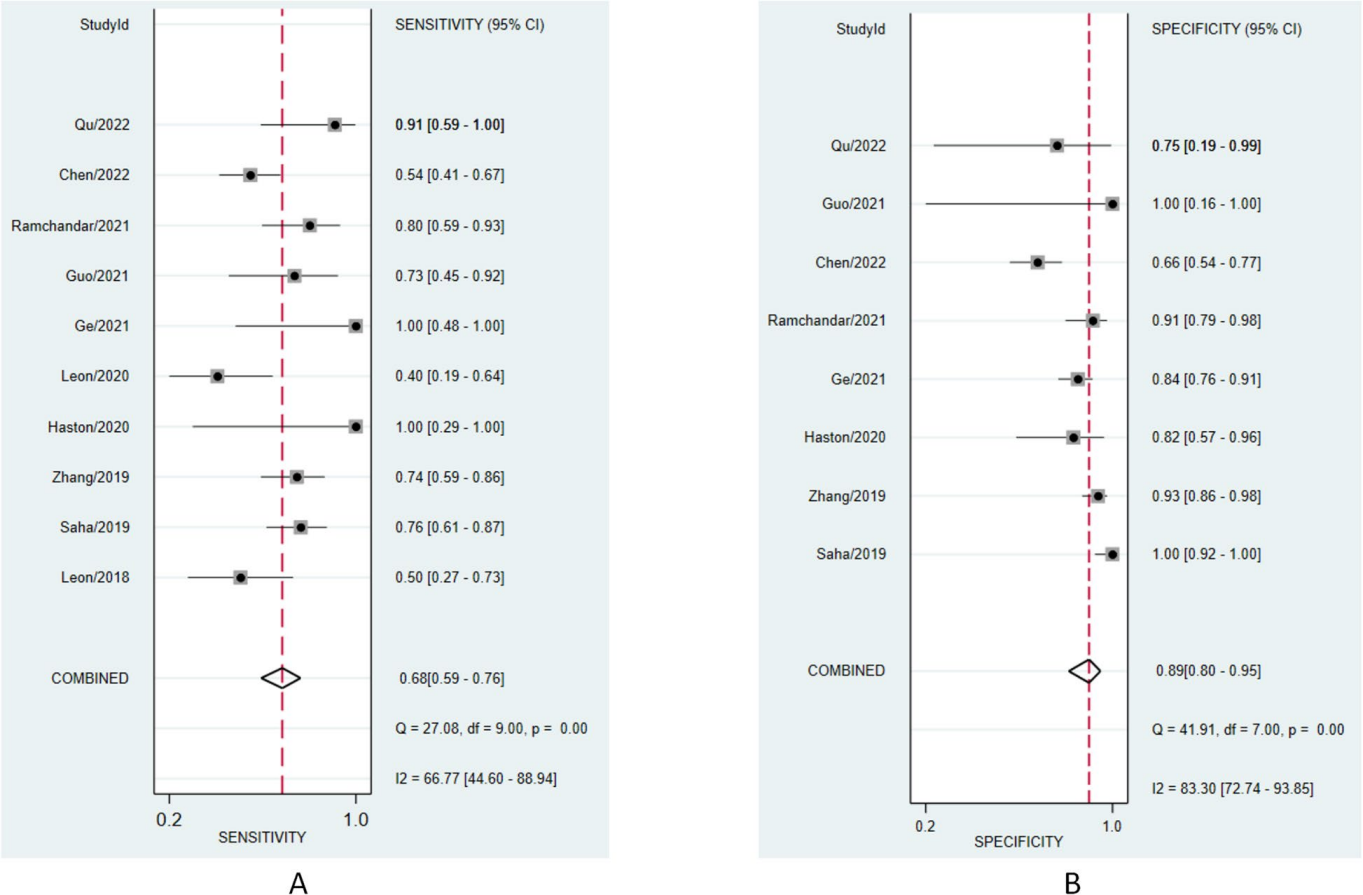
Forest plot of pooled sensitivity (**A**) and specificity (**B**) of mNGS for pediatric CNSI diagnosis

**Fig. 4:**
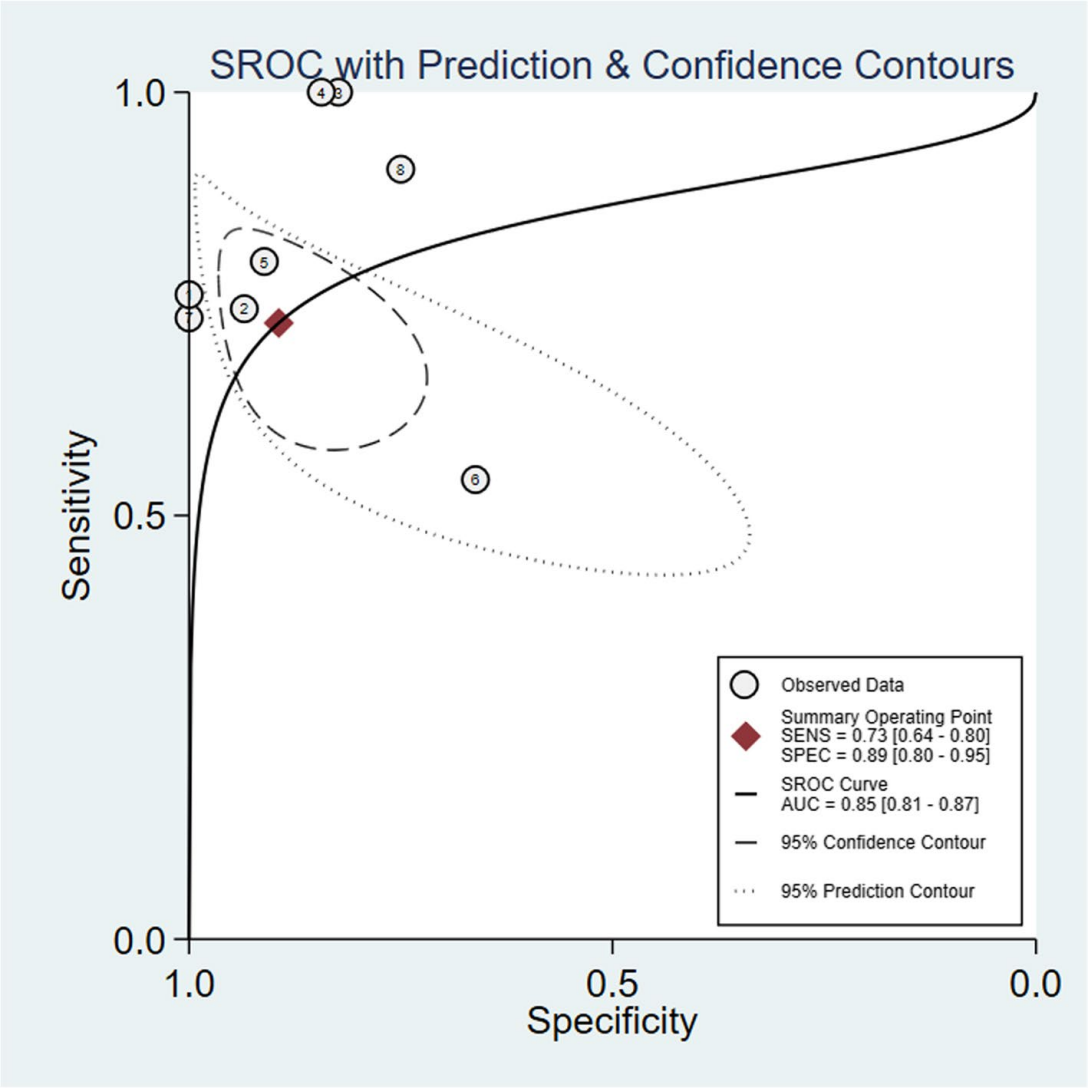
The sROC of mNGS diagnosis in pediatric CNSI

A random effect model was chosen to calculate the pooled sensitivity and specificity. The pooled sensitivity of mNGS for the diagnosis of CNSI from CSF was 0.68 (95% CI: 0.59 to 0.76, *I*^2^ = 66.77%, *p* < 0.001) (Fig. 3, A), and the pooled specificity was 0.89 (95% CI: 0.80 to 0.95, *I*^2^ = 83.37%, *p* < 0.001) (Fig. 3, B). The summary receiver operating characteristic (sROC) curve was “shoulder-arm” shaped, which indicated that there might be a potential threshold effect. The sensitivity (true-positive rate) increases with the [1-specificity] (false-positive rate). The area under the curve (AUC) for the summary receiver operating characteristic (sROC) curve was 0.85 (95% CI: 0.81 to 0.87) (Fig. 4).

The positive likelihood ratio (PLR) of mNGS ranged from 1.60 (95% CI: 1.07 to 2.41) to 69.49 (95% CI: 4.39 to 1099.58). Furthermore, the pooled PLR was 5.65 (95% CI: 2.64 to 12.10), and the *I*^*2*^ was 81.8% (*p* < 0.001) (Supplementary Fig. 2). Then, the NLR of mNGS ranged from 0.10 (95% CI: 0.01 to 1.41) to 0.69 (95% CI: 0.50 to 0.96). Furthermore, the pooled NLR was 0.29 (95% CI: 0.17–0.50), and the *I*^*2*^ was 71.2% (*p* = 0.001) (Supplementary Fig. 3). The DOR of mNGS ranged from 2.32 (95% CI: 1.13 to 4.75) to 280.91 (95% CI: 16.00 to 107.38). The pooled DOR was 26.29 (95% CI: 6.43 to 107.38), and the *I*^*2*^ was 79.7% (*p* < 0.001) (Supplementary Fig. 4).

### Publication bias

**Fig. 5:**
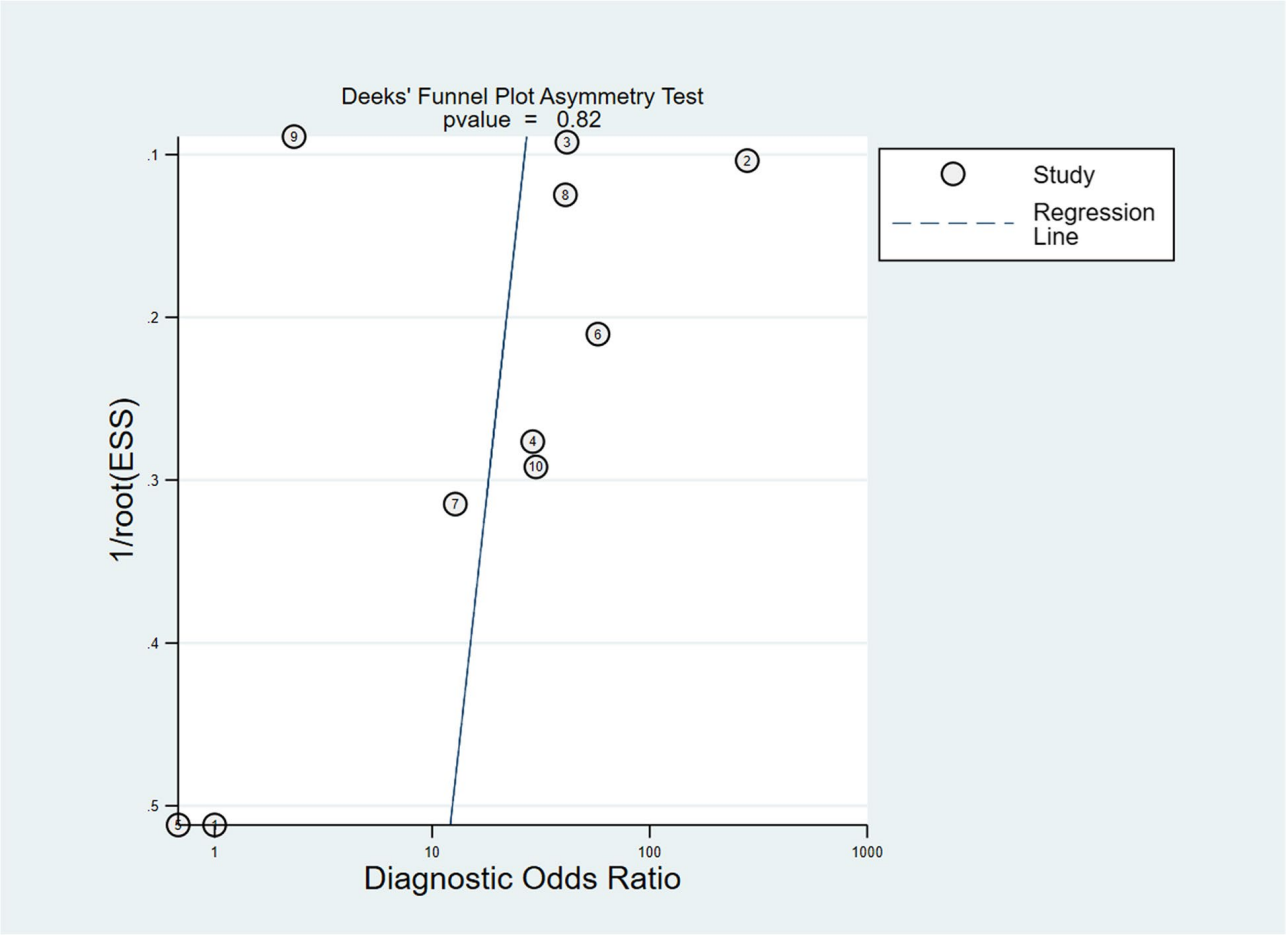
Publication bias assessed by Deek’s funnel test

Publication bias was calculated by Deek’s funnel plot asymmetry test, and the results indicated that there was no publication bias (Fig. 5, *p* = 0.82).

### Sensitivity analysis

The leave-one-out analysis indicated that the heterogeneity of pooled specificity was two studies, Chen et al. and Saha et al. [28, 30], and after removing each study, the heterogeneity was found to be 62.3% and 74.5%, respectively while no study significantly affected the heterogeneity of pooled sensitivity (Supplementary Table 1).

### Subgroup analysis

We performed subgroup analysis to investigate the source of heterogeneity. If *I*^2^ < 50%, heterogeneity in this subgroup was considered low. Among the subgroup analyses, “pathogen type” was the only factor that significantly affected heterogeneity (*p* = 0.01). Other factors were not a source of heterogeneity (*p* > 0.05 for all). Detailed information about the subgroup analysis is listed in Table 2.

**Table 2:** Subgroup and meta-regression analysis

Parameter	Subgroup	Number of studies	Sensitivity (95% CI)	*I*^2^	*p*1 (meta-regression)	Specificity (95% CI)	*I*^2^	*p*2 (meta-regression)
Continent					0.92			0.82
	Asia	6	0.72 (0.62, 0.81)	72.40%		0.91 (0.82, 0.98)	88.66%	
	Non-Asian	4	0.60 (0.45, 0.76)	71.16%		0.89 (0.75, 1.00)	0.00%	
Study direction					0.79			0.67
	Retrospective	6	0.68 (0.55, 0.81)	70.51%		0.96 (0.92, 1.00)	54.57%	
	Prospective	4	0.75 (0.59, 0.91)	80.85%		0.82 (0.73, 0.91)	75.21%	
Type of study					0.44			0.51
	Cohort	7	0.70 (0.57, 0.83)	74.50%		0.82 (0.74, 0.91)	75.21%	
	Case–control	3	0.77 (0.53, 1.00)	0.00%		0.96 (0.92, 1.00)	10.12%	
Patients were Severely ill or immunocompromised					0.17			0.18
	Yes	1	0.91 (0.94, 1.00)	/		0.77 (0.26, 1.00)	/	
	No	9	0.67 (0.58, 0.75)	68.56%		0.89 (0.82, 0.97)	86.15%	
Sample method					0.28			0.96
	Frozen	4	0.67 (0.53, 0.81)	77.03%		0.83 (0.68, 0.97)	80.59%	
	Fresh	6	0.68 (0.56, 0.80)	65.60%		0.92 (0.86, 0.98)	65.66%	
Pathogen type					0.90			0.01
	Mixed	6	0.75 (0.63, 0.87)	70.81%		0.83 (0.78, 0.88)	83.80%	
	Isolated	4	0.61 (0.45, 0.76)	26.20%		0.94 (0.87, 0.98)	0.00%	
Sequencing platform					0.87			0.79
	BGISEQ	3	0.77 (0.64, 0.90)	0.00%		0.93 (0.86, 0.97)	0.00%	
	Illumina	7	0.64 (0.53, 0.74)	70.69%		0.88 (0.74, 0.95)	86.85%	

## Discussion

mNGS has been utilized in the diagnosis of pathogens and has revealed potential value, especially in rare pathogen infections and some pathogens hard to diagnose by conventional methods (e.g., *Mycobacterium tuberculosis*) [31, 32]. Li et al. [33] indicated that mNGS may improve the accuracy of clinical diagnosis, especially in culture-negative patients. A previous study suggested that mNGS has moderate accuracy in the diagnosis of CNSI, but the study population was not limited to pediatric patients [34, 35]. According to our search results, there is no former meta-analysis related to mNGS in pediatric CNSI, and it is necessary to fill this gap.

In addition to molecular assays using specific probes or primes, mNGS detects the pathogen by characterizing all DNA or RNA in a sample, which enables the analysis of the entire microbiome [8]. mNGS mainly consists of nucleic acid extraction, library preparation, host sequence exclusion, and pathogen sequence enrichment [36]. After its first use in the detection of neuroleptospirosis in a 14­year­old patient with meningoencephalitis, mNGS has become a useful tool in infectious disease diagnosis with significant advantages [13]. According to the meta-analysis by Liu et al., mNGS presents excellent performance in infectious disease diagnosis [37].

Our study enrolled 10 studies in a meta-analysis and showed that mNGS had a high accuracy in the diagnosis of pediatric CNSI, with a combined sensitivity of 0.68, a combined specificity of 0.89, and an AUC of 0.85. The high AUC of sROC demonstrated the feasibility of mNGS in CSF for the diagnosis of pediatric CNSI. Our results are similar to the studies based on all age groups [34, 35]. However, the AUC was lower than that in these two studies (0.85 vs 0.91, both). Another meta-analysis focused on all-cause CNSI (bacteria, virus, fungal) and observed higher sensitivity and specificity of 75% and 96%, respectively [38]. The higher difficulty in obtaining samples from pediatric patients may significantly decrease the diagnostic performance of mNGS [35]. The sensitivity in the studies by Ge et al. and Haston et al. was the highest (100%). The possible reason for this is the small population in investigation and a single category of pathogens. The studies by Guo et al. and Leon et al. had the highest specificity (100%). Leon et al. only focused on encephalitis caused by enterovirus A71 without other interferences. The number of pathogens can also influence the performance of mNGS [32]. In addition, incomplete or missing descriptions of patient selection and reference standards mainly contributed to the risk of bias and applicability concerns to decrease the quality of studies.

Based on our subgroups, no significant differences were found in different subgroups from the meta-regression analysis, except the specificity in “pathogen type” (*p* < 0.05). One of the possible reasons is that the number of studies included was not large enough. However, this is also an important signal for the utilization of mNGS. For patients who may have coinfection with multiple pathogens, mNGS can be a potentially better choice to detect pathogens rapidly. Since there was only one study by Chen et al. [32] that focused on immunocompromised patients in our meta-analysis, bias may exist. Pathogen detection in immunocompromised patients is particularly challenging due to the rarity of neuroinvasive pathogens [39, 40]. As one of the major advantages of mNGS, for severely ill or immunocompromised patients who have not been diagnosed by conventional testing, it is one of the best choices to perform mNGS at that time or even earlier to improve the prognosis [41, 42]. Additionally, the different positive threshold standards may partially cause unexplained heterogeneity. It is vital to determine a positive threshold for mNGS in clinical diagnosis, but there is currently no unified international standard [32]. Furthermore, CNSI has significant regional differences, and the region of included studies was not wide enough. More multicenter trials are expected in the future [43, 44].

Pediatric CNSI is a life-threatening disease with a large population involved, causing great concern. Conventional microorganism culture is set as the gold standard, but the detection spectrum is limited, resulting in high false negativity, and the sensitivity may decline after the use of antibiotics [45, 46]. mNGS presented great potential in pathogen detection, especially in culture-negative samples. However, mNGS also has deficiencies and limitations. Based on the mechanism of mNGS, the detection results are easily affected by background interference, such as nucleic acids from hosts or the environment [47]. Additionally, mNGS can only detect pathogens without pathogen virulence and drug sensitivity, so it is poor in guiding the use of antibiotics. Moreover, the high cost is a limitation in clinical application [31]. In addition, the performance of different platforms is controversial. Zhang et al. [48] compared two platforms, Illumina and Nanopore, in broncho-alveolar lavage fluid. There was no significant difference in the coincidence rate between them. Nanopore was better in fungal detection and poor in bacterial detection with a shorter turn-around time. Compared with Illumina, BGISEQ presented a lower false rate and better sensitivity, while Illumina has the highest genome coverage [34, 49]. Currently, third-generation sequencing (TGS) is much more developed and applied in pathogen detection to fill in several gaps since it can generate long reads [50–52], but its high error rate and low accuracy are still major challenges. However, data on TGS in CSF are still lacking. Whether for NGS or TGS, the appropriate positive threshold needs to be determined urgently to improve the clinical feasibility by considering the sensitivity, specificity, biological characteristics of pathogens, and features of diseases (e.g., prevalence, harmfulness, window period) [53]. More studies are needed to unify the threshold standard for defining mNGS-positive as much as possible.

There are some strengths and limitations in the present study. To our knowledge, this is the first meta-analysis to investigate the diagnostic performance of mNGS in pediatric CNSI with a large enrolled sample size. Then, the certainty of evidence assessment was performed by the GRADE score system to enhance the reliability of this meta-analysis. However, there was significant heterogeneity among the included studies. We attempted to explore the source of heterogeneity through subgroup analysis, but the heterogeneity of each subgroup did not significantly decrease. The possible causes of clinical heterogeneity were analyzed as follows. First, some studies did not describe clear diagnostic standards for CNSI. Second, several studies were unable to distinguish encephalitis or meningitis. However, due to the limited number of studies included, we could not conduct a more in-depth analysis, which may affect the accuracy of the results, more studies are expected.

## Conclusion

mNGS has a good diagnostic performance for CSF pathogens in pediatric CNSI patients. For pediatric patients with unclear diagnosis or severe illness, mNGS is the best choice to obtain a diagnosis for future treatment. Although there are still some limitations in mNGS, such as high cost and inconsistency of the positive threshold, we still believe that with deeper research and continuous technical improvement in mNGS, the diagnostic performance will be better in the future, and the application of mNGS will be more common.

### Supplementary Information

**Additional file 1: Supplementary file.** The command used in Stata Software. **Supplementary Figure 1.** The certainty of evidence measure by GRADE score system. **Supplementary Figure 2.** Forest plot for the positive likelihood ratio (PLR) of mNGS for the diagnosis of pediatric CNSI. **Supplementary Figure 3.** Forest plot for the negative likelihood ratio (NLR) of mNGS for the diagnosis of pediatric CNSI. **Supplementary Figure 4.** Forest plot for the Diagnostic Odd’s Ratio (DOR)** Supplementary Table 1.** Leave-one-out analysis depicting the pooled sensitivity and specificity.

## Data Availability

Not applicable.

## Abbreviations

AUC: Area under curve
CNSI: Central nervous system infection
CI: Confidence interval
CSF: Cerebrospinal fluid
DOR: Diagnostic odd's ratio
FN: False negative
FP: False positive
GRADE: Grading of Recommendations, Assessment, Development, and Evaluations
mNGS: Metagenomic next-generation sequencing
NLR: Negative likelihood ratio
PLR: Positive likelihood ratio
QUADAS: Quality Assessment of Diagnostic Accuracy Studies
sROC: Summary receiver operating characteristic
TGS: Third-generation sequencing
TN: True negative
TP: True positive
