# Supplementary material for: Diagnostic performance of metagenomic next-generation sequencing for the detection of pathogens in cerebrospinal fluid in pediatric patients with central nervous system infection: a systematic review and meta-analysis
Source: BMC Infect Dis. 2024 Jan 18;24:103. doi: 10.1186/s12879-024-09010-y (PMC10797782; doi:10.1186/s12879-024-09010-y)
Supplement: Supplementary file 1 — Additional file 1: Supplementary file. The command used in Stata Software. Supplementary Figure 1. The certainty of evidence measure by GRADE score system. Supplementary Figure 2. Forest plot for the positive likelihood ratio (PLR) of mNGS for the diagnosis of pediatric CNSI. Supplementary Figure 3. Forest plot for the negative likelihood ratio (NLR) of mNGS for the diagnosis of pediatric CNSI. Supplementary Figure 4. Forest plot for the Diagnostic Odd’s Ratio (DOR) Supplementary Table 1. Leave-one-out analysis depicting the pooled sensitivity and specificity. [file 12879_2024_9010_MOESM1_ESM.docx]

**Supplementary file**

**The command used in Stata Software:**

ssc install midas

ssc install mylabels

midas tp fp fn tn, res(sum)

midas tp fp fn tn, id(author year) ms(0.75) ford fors bfor(dss)

midas tp fp fn tn, plot sroc(both)

midas tp fp fn tn, pubbias


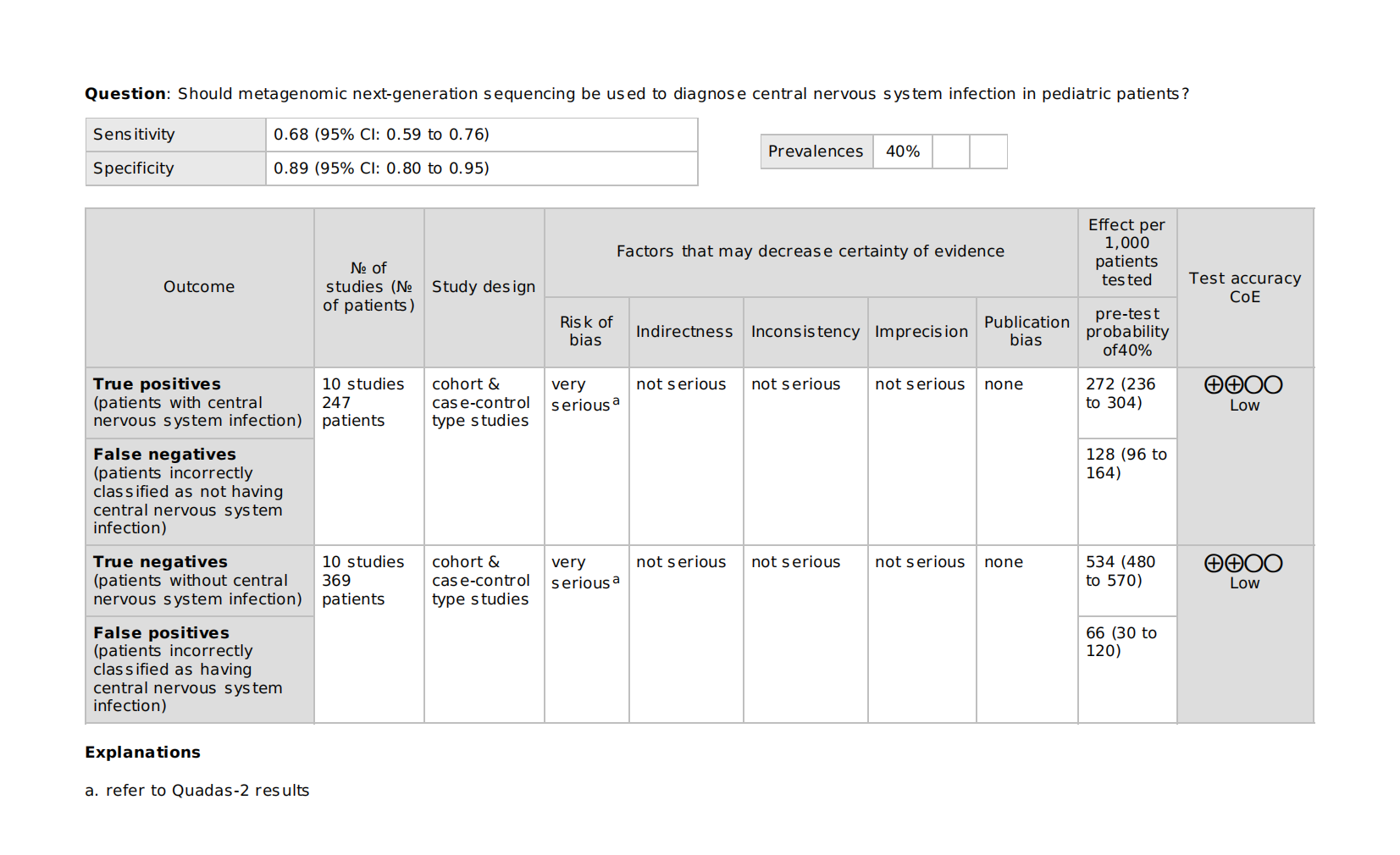


**Supplementary Figure 1:** The certainty of evidence measure by GRADE score system


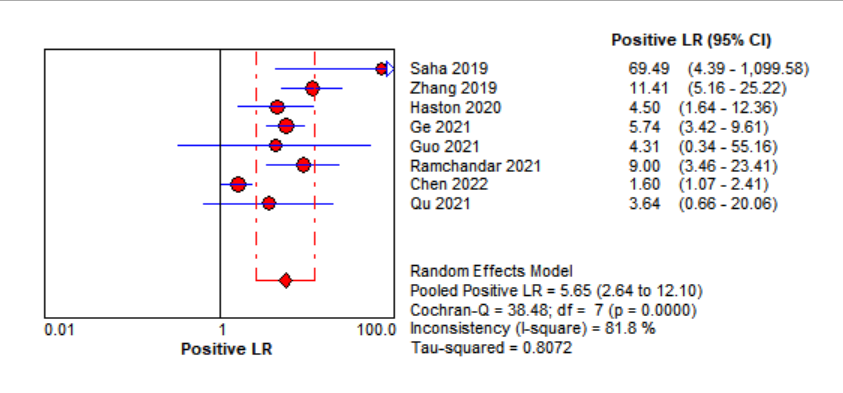


**Supplementary Figure 2:** Forest plot for the positive likelihood ratio (PLR) of mNGS for the diagnosis of pediatric CNSI.


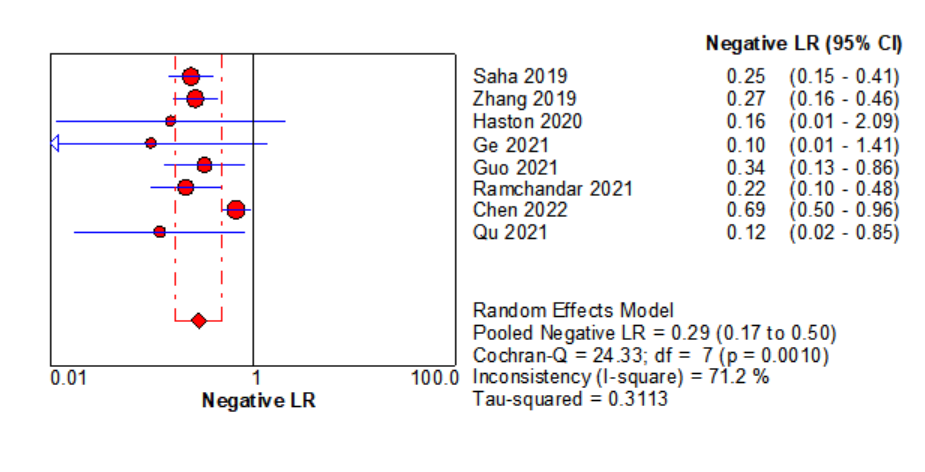


**Supplementary Figure 3:** Forest plot for the negative likelihood ratio (NLR) of mNGS for the diagnosis of pediatric CNSI.


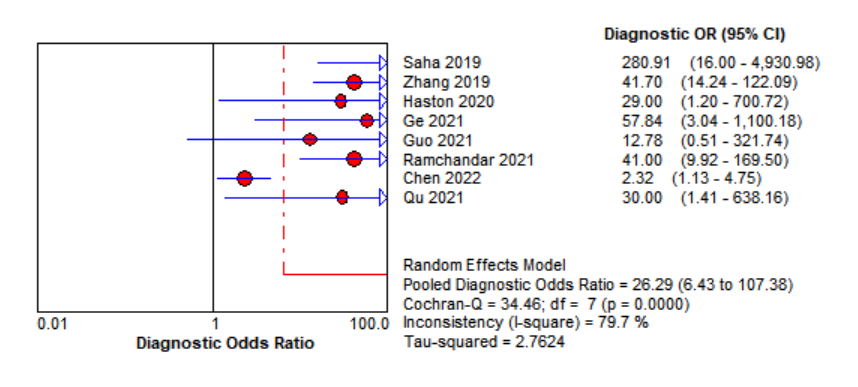


**Supplementary Figure 4:** Forest plot for the Diagnostic Odd’s Ratio (DOR)

**Supplementary Table 1:** Leave-one-out analysis depicting the pooled sensitivity and specificity

| Sensitivity | | | Specificity | | |
| --- | --- | --- | --- | --- | --- |
|  | Estimate (95% CI) | *I^2^* value |  | Estimate (95% CI) | *I^2^* value |
| **Overall** | 0.68 (0.59, 0.76%) | 66.8% | **Overall** | 0.89 (0.80, 0.95) | 83.3% |
| Omitted Study |  |  | Omitted Study |  |  |
| (Qu, 2022) | 0.66 (0.60, 0.72) | 67.1% | (Qu, 2022) | 0.86 (0.82, 0.89) | 84.1% |
| (Chen, 2022) | 0.71 (0.64, 0.78) | 64.0% | (Chen, 2022) | 0.90 (0.86, 0.93) | 62.3% |
| (Ramchandar, 2021) | 0.66 (0.59, 0.72) | 68.8% | (Ramchandar, 2021) | 0.85 (0.81, 0.89) | 83.7% |
| (Guo, 2021) | 0.67 (0.60, 0.73) | 71.0% | (Guo, 2021) | 0.86 (0.82, 0.89) | 84.0% |
| (Ge, 2021) | 0.67 (0.60, 0.72) | 66.5% | (Ge, 2021) | 0.86 (0.82, 0.90) | 84.2% |
| (Leon, 2020) | 0.70 (0.63, 0.76) | 62.1% | (Haston, 2020) | 0.86 (0.82, 0.90) | 84.2% |
| (Haston, 2020) | 0.67 (0.61, 0.73) | 68.6% | (Zhang, 2019) | 0.83 (0.79, 0.88) | 81.0% |
| (Zhang, 2019) | 0.66 (0.59, 0.72) | 70.0% | (Saha, 2019) | 0.84 (0.80, 0.88) | 74.5% |
| (Saha, 2019) | 0.65 (0.58, 0.72) | 69.0% |  |  |  |
| (Leon, 2018) | 0.69 (0.62, 0.75) | 68.2% |  |  |  |
